# Functional and Biochemical Characterization of Human Eukaryotic Translation Initiation Factor 3 in Living Cells

**DOI:** 10.1128/MCB.00663-14

**Published:** 2014-08

**Authors:** Susan Wagner, Anna Herrmannová, Radek Malík, Lucie Peclinovská, Leoš Shivaya Valášek

**Affiliations:** a Laboratory of Regulation of Gene Expression, Institute of Microbiology ASCR, Videnska, Prague, Czech Republic; b Laboratory of Epigenetic Regulations, Institute of Molecular Genetics ASCR, Videnska, Prague, Czech Republic

## Abstract

The main role of the translation initiation factor 3 (eIF3) is to orchestrate formation of 43S-48S preinitiation complexes (PICs). Until now, most of our knowledge on eIF3 functional contribution to regulation of gene expression comes from yeast studies. Hence, here we developed several novel *in vivo* assays to monitor the integrity of the 13-subunit human eIF3 complex, defects in assembly of 43S PICs, efficiency of mRNA recruitment, and postassembly events such as AUG recognition. We knocked down expression of the PCI domain-containing eIF3c and eIF3a subunits and of eIF3j in human HeLa and HEK293 cells and analyzed the functional consequences. Whereas eIF3j downregulation had barely any effect and eIF3a knockdown disintegrated the entire eIF3 complex, eIF3c knockdown produced a separate assembly of the a, b, g, and i subunits (closely resembling the yeast evolutionary conserved eIF3 core), which preserved relatively high 40S binding affinity and an ability to promote mRNA recruitment to 40S subunits and displayed defects in AUG recognition. Both eIF3c and eIF3a knockdowns also severely reduced protein but not mRNA levels of many other eIF3 subunits and indeed shut off translation. We propose that eIF3a and eIF3c control abundance and assembly of the entire eIF3 and thus represent its crucial scaffolding elements critically required for formation of PICs.

## INTRODUCTION

Eukaryotic translation initiation factor 3 (eIF3) is a highly complex multiprotein assembly with multiple functions in translation. It promotes formation of 43S and 48S preinitiation complexes (PICs) by aiding the Met-tRNA_i_^Met^ and mRNA loading onto the small ribosomal subunit, and, at least in the budding yeast, it was also implicated in stimulating subsequent scanning for AUG recognition (for a review, see reference 1). The complexity of this factor is also reflected by a variety of processes that it is involved in beyond initiation. In particular, mammalian eIF3 was suggested to act during ribosomal recycling *in vitro* (2), and budding yeast eIF3 and one of its associated factors HCR1 have only recently been implicated in controlling translation termination and stop codon readthrough *in vivo* (3). Moreover, eIF3 was demonstrated to link translation initiation to transcription (4), to mRNA export (5), and to the nonsense-mediated decay pathway (6). In addition to its involvement in regulation of cellular gene expression, eIF3 also stimulates translation of hepatitis C virus (HCV) proteins (7) and, unexpectedly, may even act to inhibit HIV replication as part of the antiviral self-defense mechanism (8). With such a broad spectrum of functions, it is no surprise that misregulation of expression of several eIF3 subunits has been implicated in oncogenesis and the maintenance of a cancerous state (9).

**FIG 1:**
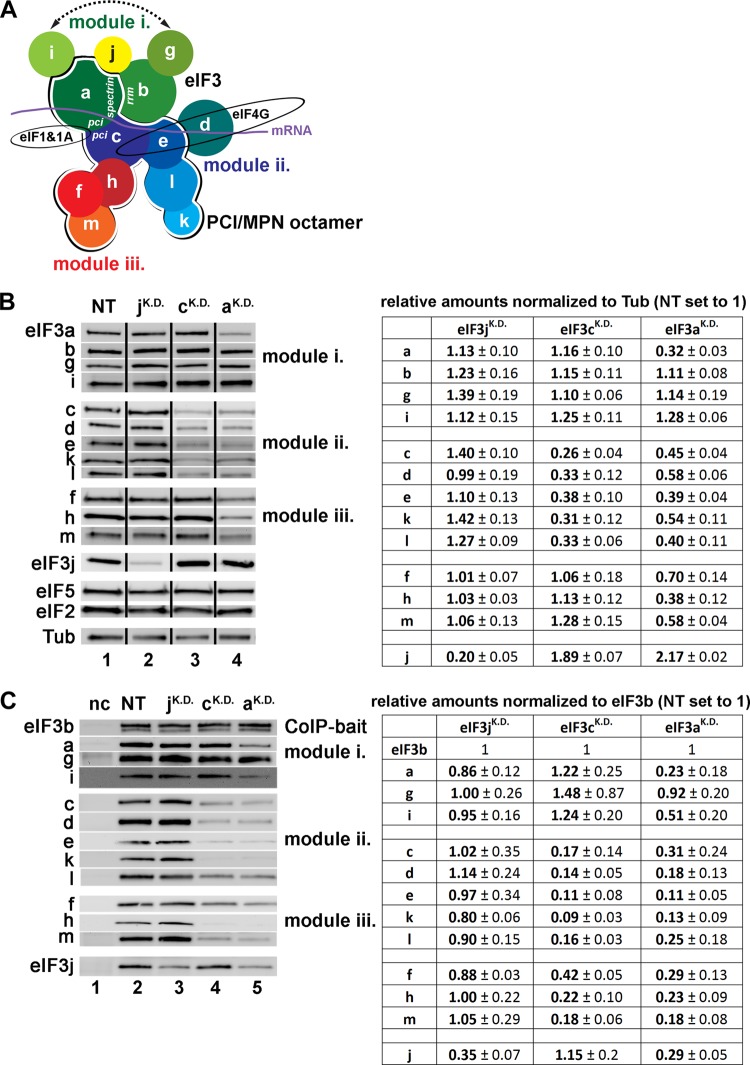
eIF3c and eIF3a control expression of module ii and iii subunits of eIF3 and link all three modules tightly together in HeLa cells. (A) A schematic model of human eIF3 and its binding partners combining findings presented here and elsewhere (7, 10, 13, 14, 26, 46). Individual eIF3 modules as well as eIF3 subunits forming the PCI/MPN octamer are indicated. An arrow indicates a potential interaction between eIF3i and -g as proposed in reference 10. (B) Effect of siRNA targeting eIF3j (2 nM), eIF3c (5 nM), and eIF3a (5 nM) mRNAs on protein levels of eIF3 subunits and other eIFs determined by Western blotting 72 h posttransfection (this experiment was repeated numerous times with similar results). NT, control nontargeted cells; Tub, loading control, anti-TUBA4A from Sigma, catalog no. T6074. WCEs were prepared as described in Materials and Methods. Western blots were quantified using Quantity One and/or NIH ImageJ, and the signals were normalized to α-tubulin (Tub). The resulting values obtained with the control NT cells were set to 1.00, and those obtained with eIF3j^KD^, eIF3c^KD^, and eIF3a^KD^ cells were expressed relative to the NT. Standard deviations (SD) from at least three individual experiments are given. (C) Effect of siRNA targeting eIF3j (2 nM), eIF3c (5 nM), and eIF3a (5 nM) mRNAs on integrity of eIF3 determined by the coimmunoprecipitation assay using eIF3b as a co-IP bait (anti-eIF3b antibody from Santa Cruz, catalog number sc-16377) followed by Western blotting 72 h posttransfection (see also Fig. S3 in the supplemental material). nc, negative control (beads only, no antibody). Western blots were quantified using Quantity One and/or NIH ImageJ, and the signals were normalized to eIF3b. The resulting values obtained with the control NT cells were set to 1.00, and those obtained with eIF3j^KD^, eIF3c^KD^, and eIF3a^KD^ cells were expressed relative to the NT (SD are given).

In the past decade, a considerable effort has been put into elucidation of the composition and structure of mammalian eIF3, containing altogether 12 major subunits referred to here as the eIF3 holocomplex (10). First, the low-resolution cryoelectron microscopy (cryo-EM) structure of purified endogenous ∼800-kDa human eIF3 revealed a five-lobed structure with anthropomorphic features (11). Subsequently, a biochemical study suggested that the functional core of mammalian eIF3 comprises the a, b, c, e, f, and h subunits (12). Later, tandem mass spectrometry work identified three stable modules of human eIF3: i (composed of a, b, g, and i subunits, whose Saccharomyces cerevisiae homologues, together with the homologue of eIF3c, form the yeast 5-subunit eIF3 [1]), ii (formed by c, d, e, k, and l subunits with the e:l:k trimer adopting an elongated, linear topology), and iii (subunits f, h, and m with a compact trigonal geometry) (10, 13) (Fig. 1A). The eIF3c subunit was proposed to link modules i and iii by simultaneous binding to their a/b and h subunits, respectively. More recently, several cryo-EM studies shed more light on the spatial arrangement of mammalian eIF3, proposing that its core is actually formed by the PCI/MPN octamer, including subunits a (lacking its C-terminal half), c (lacking its N-terminal one-third), and e, f, h, k, l, and m (14–16) (Fig. 1A). The PCI/MPN octamer represents a specific protein assembly of 6 subunits carrying the PCI (for proteasome, COP9, eIF3) domain and 2 additional subunits carrying the MPN (for Mpr1-Pad1 N-terminal) domain, the compositional arrangement of which is shared with two other functionally unrelated multiprotein complexes: the COP9 signalosome and the 19S proteasome lid (17). It should be mentioned here that the existence of the eIF3 PCI/MPN octamer is compatible with the earlier proposed modularity of eIF3, as the b, i, and g subunits may adhere to the octamer by stable binding to its a subunit (together forming module i), and the d subunit may associate with the e subunit of the octamer (forming module ii), with module iii being already an integral part of the eIF3 octamer (Fig. 1A). Surprisingly, the three-dimensional (3D) reconstruction of a bacterially expressed octameric core complex was very similar to the 3D structure of the 12-subunit eIF3 holocomplex, suggesting that the remaining 4 subunits (b, g, i, and d) are rather flexible within the entire structure (15).

Whereas mutual intersubunit contacts of yeast eIF3 as well as its contacts with other eIFs and the 40S ribosome have been extensively mapped (see, for example, references 1 and 18–20), a comprehensive subunit interaction map for mammalian eIF3 and its interacting partners has not been reported yet. Among a very few examples of characterized interactions, two recently identified RNA binding domains of eIF3a and eIF3c were shown to mediate eIF3 binding to the 40S subunit and to the HCV mRNA internal ribosome entry site (IRES) and thus promote HCV translation (7). It is highly likely that under standard conditions, these two subunits may promote mRNA recruitment to the 43S PICs, as was proposed for their yeast counterparts TIF32 and NIP1 (21–23). Interestingly, eIF3a and eIF3c were also proposed to interact with eIFs 1 and 1A (14) (Fig. 1A). Recently, the spectrin domain of eIF3a was suggested to serve as the docking site for formation of the eIF3 module i (a, b, g, i). It directly and concurrently interacted with eIF3b and eIF3i, whereas its association with eIF3g was mediated by the C-terminal domain (CTD) of eIF3b (24). The binding of eIF3b to the spectrin domain of eIF3a occurred through its RNA recognition domain (RRM), in agreement with our observations in yeast (25). In contrast to yeast, however, eIF3a interacted with the eIF3b-RRM in a mutually exclusive manner with eIF3j (HCR1 in yeast). The authors proposed that eIF3a organizes the module i via its spectrin domain and connects it with the PCI/MPN octamer via the PCI-PCI interaction with eIF3c (Fig. 1A). Finally, Fraser's group recently reported that human eIF4G has two distinct binding sites for eIF3, one of which interacts with eIF3c and -d subunits, whereas the other binds eIF3e (26). These interactions were proposed to promote mRNA binding to the 40S ribosome in the eIF4G-dependent manner (Fig. 1A).

The aforementioned eIF3j protein has been considered the 13th, only loosely associated, eIF3 subunit, which is often missing from the purified 12-subunit complex and whose placement in the entire assembly map of mammalian eIF3 is rather unclear (10, 12, 14, 15, 27). eIF3j was shown to associate with the 40S ribosome independently of the rest of eIF3 and critically promote binding of eIF3 to purified 40S subunits *in vitro* or to bare 40S subunits in quiescent T lymphocytes upon their activation (28, 29). At the same time, however, it was paradoxically shown to be dispensable for formation of the 43S-48S PICs in *in vitro*-reconstituted mammalian systems (12, 27). eIF3j was suggested to reside near the ribosomal A site, where it may interact with eIF1A and prevent premature recruitment of mRNA (30). Finally, it was also proposed to promote mRNA dissociation during the ribosomal recycling step (2). In contrast, its yeast homologue, HCR1, is nonessential (31), its contribution to 40S binding by eIF3 *in vivo* is less critical (32, 33), and, most importantly, it has only recently been implicated in controlling the translation termination and stop codon readthrough as its major role in the cell (3). Owing to that, it was suggested to consider HCR1 as an independent initiation factor that associates and closely cooperates with the eIF3 holocomplex but is not its integral part (3). It remains to be seen whether the same holds true also for eIF3j. In any case, the observed discrepancy between the importance of yeast HCR1 and its human ortholog eIF3j for cell proliferation has been rather puzzling.

Despite the recent progress, our understanding of the roles of individual human eIF3 subunits in initiation is limited because of the scarcity of functional studies employing the *in vivo* approaches. Here, we established several such assays and knocked down expression of eIF3c, eIF3a, and eIF3j in HeLa and HEK293 cells. We observed that downregulation of eIF3j leads to a very modest effect on efficiency of protein synthesis and practically no effect on integrity of the eIF3 holocomplex and its 40S association. In contrast, knocking down eIF3c or eIF3a is deleterious, as it dramatically affects expression and/or stability of many other eIF3 subunits, impairs assembly of the eIF3 holocomplex, and, consequently, interferes with eIF3 binding to the 40S ribosome as well as with mRNA recruitment and fidelity of AUG recognition *in vivo*. Based on our findings, we conclude that whereas eIF3j has most likely only a supportive role in the overall translational efficiency in human cells, eIF3c and eIF3a subunits of the PCI/MPN octamer are critically required for expression and integrity of the entire eIF3 holocomplex and thus for assembly of the 43S-48S PICs in general. Especially eIF3a seems to hold a strict control over abundance of most of the other eIF3 subunits, which may make it a promising target in anticancer therapies.

## MATERIALS AND METHODS

### WCE preparation.

Cells were grown in 24-well plates or in 15-cm dishes in Dulbecco's modified Eagle's medium (DMEM) (Sigma; catalog no. D6429) supplemented with 10% fetal bovine serum (FBS) (Sigma; catalog no. F7524) to ∼80% confluence, washed with cold 1× phosphate-buffered saline (PBS), and lysed directly on a plate in lysis buffer A (20 mM Tris-HCl [pH 7.5], 50 mM KCl, 10 mM MgCl_2_, 5 mM NaF, 1 mM dithiothreitol [DTT], 1 mM phenylmethylsulfonyl fluoride (PMSF), 1 μg/ml aprotinin, 1 μg/ml leupeptin, 1 μg/ml pepstatin, 1 Mini Complete EDTA-free (Roche) tablet/5 ml, 1% Triton X-100). Lysate was then collected in centrifugation tubes by scraping and pipetting, and, after a 5-min incubation on ice with occasional vortexing, cleared by centrifugation at 13,000 rpm for 5 min at 4°C. Generated whole-cell extracts (WCEs) were then used for Western blot analysis.

### Western blot quantifications.

Western blotting signal was developed using SuperSignal West Femto maximum sensitivity substrate from Thermo Scientific (catalog no. 34096) and the G-Box imager from Syngene in a series of various exposure times. Thus, obtained signals were processed with Quantity One (Bio-Rad) and ImageJ 1.42q (NIH). Only signals from the same strips and with the same exposure time were compared. At least three individual experiments were used for final calculations. The resulting values were normalized as indicated in the corresponding figure legends. Thus, obtained values for the control nontargeting (NT) cells were set to 1.00 and those obtained for eIF3j^KD^, eIF3c^KD^, and eIF3a^KD^ cells were expressed relative to the NT with standard deviations (SD).

### Transfections.

Twenty-four hours after seeding, the cells were transfected with ON-TARGETplus small interfering RNA (siRNA) cocktail systems from Dharmacon/Thermo Scientific (human eIF3j, catalog no. L-019532-00; human eIF3c, catalog no. L-009036-00; human eIF3a, catalog no. L-019534-00; nontargeting siRNA, catalog no. D-001810-10) at the concentrations mentioned in the figures. The nontargeting siRNA was applied at the highest concentration of a given siRNA on target. INTERFERin (Polyplus; catalog no. 409) was used as a transfection reagent, and transfection was carried out according to the vendor's instructions (3 μl/well of a 24-well plate, 100 μl/15-cm dish).

For both the Sui^−^ and leaky scanning assays, transfection of the reporter plasmids was carried out 48 h after siRNA transfection using 0.5 μl TurboFect (Thermo Scientific; catalog no. R0531) according to the vendor's instructions. In particular, for the Sui^−^ assay, 50 ng of pGL4-CMV (where CMV is cytomegalovirus) (firefly luciferase; with AUG or GUG as the start codon [this study]) combined with 50 ng of phRL-CMV (Renilla luciferase [this study]) was cotransfected; for the leaky scanning assay, 250 ng of pTK-ATF4-deltaORF1 combined with 2 ng of pSV40-Renilla luciferase (34) was cotransfected.

pGL4-CMV with AUG as the start codon was created by inserting the BglII-HindIII fragment from pcDNA3.1^−^ (Life Technologies) containing the CMV promoter into pGL4.10 (Promega).

pGL4-CMV with GUG as the start codon was created using a QuikChange multisite-directed mutagenesis kit (Stratagene; catalog no. 200514) using primers SW1 (5′ GGTAAAGCCACCGTGGAAGATGCC 3′) and SW2 (5′ GGCATCTTCCACGGTGGCTTTACC 3′).

phRL-CMV was created by exchanging the SV40 promoter from phRL-SV40 (Promega) with the CMV promoter from pcDNA3.1^−^ (Life Technologies) using BglII and NheI.

### Coimmunoprecipitation assays.

Protein A/G-agarose (Pierce; catalog no. 20421) was preincubated with anti-eIF3b (Santa Cruz; catalog no. sc-16377) for 2 h while rotating at 4°C (around 2 μg of the antibody per 100 μl of the 50% protein A/G slurry in buffer A without protease inhibitors and Triton X-100). For the negative control, incubation was carried out without antibodies. Preincubated agarose beads were washed several times with buffer A, WCE diluted in buffer A was added (∼1 mg of total protein with the final concentration of Triton X-100 at ∼0.3%), and the mixture was incubated overnight at 4°C while rotating. Subsequent washing steps were performed with buffer A containing ∼0.3% of Triton X-100. Coimmunoprecipitated (co-IP) proteins were eluted in a denaturing loading buffer by boiling, and the eluate was analyzed by SDS-PAGE followed by Western blotting. Pretreatment of cells with a cross-linking agent (HCHO) had no visible effect on the final outcome and thus was not routinely used.

### FACS analysis.

Cells were grown and transfected as usual. Three days after transfection, cells were collected with trypsin (0.25% in PBS for HeLa, 0.025% in PBS for HEK) and washed twice in PBS. A 100-μl aliquot of 200 cells/μl in the annexin buffer was stained with 20 μl of Hoechst 33258 (10 μg/ml; Sigma; catalog no. 861405) and 1 μl of annexin V-fluorescein isothiocyanate (FITC) (Apronex; catalog no. ANXV-FT100). After 3 min of incubation, cells were analyzed by fluorescence-activated cell sorting (FACS; LSR II from Becton, Dickinson; FlowJo from Tree Star Inc.). As a positive control of ongoing apoptosis, a fraction of cells was not transfected but instead treated with 0.2 μM bortezomib (Velcade) for 20 h prior to harvesting. For HEK293 cells, untransfected and untreated cells were also included as a negative control.

### MTT assay.

Cells were grown in 24-well plates. At the indicated days after siRNA transfections, medium was removed by suction and a 5:1 mixture of DMEM-10% FBS with the microculture tetrazolium (MTT) solution (5 mg/ml thiazolyl blue tetrazolium bromide in 1× PBS; Sigma; catalog no. M2128) was added and the cells were incubated for 3.5 h. Subsequently, the mixture was removed and 200 μl of the MTT solvent (4 mM HCl and 0.1% IPEGAL dissolved in isopropanol) was added and kept at room temperature for several minutes. Cells were then carefully dissolved by pipetting, and 150 μl of the resulting mixture was transferred into a flat-bottom transparent 96-well plate. Finally, absorbance at a λ of 595 nm with a reference filter λ of 620 nm was measured.

### [^35^S]methionine incorporation assay.

Cells were grown in 24-well plates. Three days after the siRNA transfections, medium was removed and fresh methionine-free DMEM was added (Sigma; catalog no. D0422; supplemented with 4 mM l-glutamine). After 1.5 h, 2 μCi of [^35^S]methionine per 100-μl final volume was added and the cells were incubated for 3 h. WCEs were prepared as described above. Cells from two wells were combined for each reaction. A total of 50 μg of protein was transferred into a new tube, and proteins were precipitated with 100% trichloric acid for at least 10 min at 4°C. Collected pellets were then washed 3 times with 1 ml of ice-cold acetone, dried, dissolved in 100 μl of 1 N NaOH by vortexing, and finally transferred into a scintillation vial, to which 150 μl of 1 N HCl and a scintillation liquid were added. Radioactivity was measured in a scintillation counter (Beckman LS6500).

### Polysome profile analysis.

Cells were grown in 15-cm dishes. Three days after siRNA transfections, cycloheximide was added to a final concentration of 100 μg/ml 1 min prior to harvesting. Cells were washed with 1× PBS and lysed in buffer A (both supplemented with 100 μg/ml cycloheximide). The resulting lysate was collected and cleared by centrifugation. Fifteen *A*_260_ units of WCE was then separated by high-velocity sedimentation through a 5% to 45% sucrose gradient at 39,000 rpm for 2.5 h using the SW41Ti rotor. The gradients were scanned at 254 nm to visualize the ribosomal species. For the calculation of the polysome-to-monosome ratio (P/M), the area under the curve corresponding to the 80S monosomes or the polysomes was quantified with the Engauge software.

### 40S binding analysis.

Cells were grown in 15-cm dishes and fixed with 0.2% formaldehyde (HCHO) for 5 min prior to harvesting and processed as described above. Ten *A*_260_ units of WCE was then separated by high-velocity sedimentation through a 7% to 30% sucrose gradient at 41,000 rpm for 5 h using the SW41Ti rotor as described previously (35). Fractions of 600 μl were collected and precipitated with 100% ethanol overnight at −20°C, and after a single washing step with 100% ethanol, the pellet was dried, dissolved in 1× loading buffer, boiled to reverse cross-linking, and analyzed by SDS-PAGE followed by Western blotting.

### mRNA binding analysis.

Cells were grown in 15-cm dishes and processed as described for the 40S binding analysis except that HCHO was not used to fix the cells. Fractions of 700 μl were collected and total RNA was isolated by phenol-chloroform extraction. After DNase I digestion (NEB; catalog no. M0303S), reverse transcription was carried out (Applied Biosystems; catalog no. 4368814) and the levels of 18S rRNA and *RPL41* mRNA were analyzed with quantitative PCR in triplicates as described before (23) (Fermentas; catalog no. K025; Roche LC480II cycler).

### Dual luciferase assay.

Cells were grown in 24-well plates. One day after the reporter plasmid transfection, the cells were washed in 1× PBS and lysed directly on a plate with 1× Glo lysis buffer (Promega; catalog no. E266A). The lysate was then transferred into a white flat-bottom 96-well plate. The Dual-Glo luciferase assay system (Promega; catalog no. E2940) was employed according to the vendor's instructions. The Renilla luciferase signal served for normalization.

## RESULTS

### eIF3c and eIF3a control expression of module ii and iii subunits of eIF3 and are critical for the integrity of the entire eIF3.

In order to examine the physiological importance of the two major scaffolding eIF3 subunits (eIF3a and eIF3c) as well as that of the puzzling eIF3j for the overall cellular proliferation, we individually knocked down all three of them in HeLa and HEK293 cells. We did that using the ON-TARGETplus SMART pool siRNA system (Dharmacon/Thermo Scientific) as described in Materials and Methods. Both cell lines were transfected with siRNAs 24 h after seeding. All experiments were performed 3 days after siRNA transfection, and we always verified the degree of downregulation by Western blotting; in all cases, we routinely achieved ∼75% reduction in protein levels of a subunit on target for HeLa and ∼60% for HEK293 cells (Fig. 1B; see also Fig. S1A in supplemental material). Generally, less efficient downregulation obtained in HEK293 compared to that in HeLa cells (that could not be increased by transfection optimization) is most probably due to differences in RNA interference (RNAi) pathway activity in these cell lines (36). Owing to that, all effects observed in HEK293 cells, which are presented below, are similar but less pronounced than those in HeLa cells.

Strikingly, by probing for other eIF3 subunits, we noticed that whereas the eIF3j knockdown (eIF3j^KD^) left the protein levels of all other eIF3 subunits unchanged, the eIF3c^KD^ dramatically reduced the protein levels of all module ii subunits (d, e, k, l) in HeLa (Fig. 1B) and HEK293 cells (see Fig. S1A in supplemental material) by ∼70% and ∼60%, respectively. The eIF3a^KD^ then severely (by ∼50%) reduced protein levels of all module ii subunits and strikingly also module iii subunits (f, h, m; by ∼45%) but not the levels of the other module i subunits, eIF3j and two other eIFs (2 and 5), which occur together with eIF3 in the so-called multifactor complex (37). Considering that mRNA levels of affected eIF3 subunits did not change relative to the control nontargeting (NT) cells (see Fig. S2 in the supplemental material), we conclude that eIF3c controls the abundance of module ii subunits and eIF3a controls the abundance of eIF3c and module iii subunits on either the translational or posttranslational level.

Next, we investigated the integrity of the eIF3 complex in all three siRNA knockdowns by performing coimmunoprecipitation experiments with anti-eIF3b antibody in both cell lines. As shown in Fig. 1C and in Fig. S3 and S1B in the supplemental material, eIF3j^KD^ had no effect, whereas the eIF3c^KD^ split eIF3 apart so that only intact module i could be retrieved from WCEs with anti-eIF3b antibody to the levels comparable to the NT control (all three subunits of module iii accumulated in “Wash” fractions [see specifically Fig. S3 in the supplemental material]). This result suggests that three eIF3 subspecies occur in eIF3c^KD^ cells: the complete 13-subunit eIF3 complex (most likely containing all that is left from the remaining amounts of module ii subunits in the cytoplasm) and separate modules i and iii. Although we cannot rule out that module iii falls apart (none of the anti-eIF3f, -h, or -m antibodies worked as “bait” in our coimmunoprecipitation assays to isolate module iii in separation in eIF3c^KD^ cells), below we present an *in vivo* indication supporting our assumption that module iii in fact holds together and does not associate only with module i (see Fig. 3). In further support, all three eIF3f, -h, and -m subunits were shown to form a stable heterotrimer both *in vitro* and *in vivo* (10, 38).

The eIF3a^KD^ had in principle the same effect as eIF3c^KD^, but on top of it, it further disintegrated module i. As a result, only eIF3g was retrieved using eIF3b as bait with wild-type (WT) efficiency (Fig. 1C). In accord, previous analysis showed that eIF3 lacking eIF3a does not assemble at all *in vitro* (12). Also note that the amounts of eIF3j were likewise markedly reduced, which is in contrast with the recent *in vitro* observation suggesting that eIF3j and eIF3a interact with the RRM of eIF3b in a mutually exclusive fashion (24). In fact, from our data, it seems that eIF3a and not eIF3b is the main anchor for eIF3j in the eIF3 complex. Finally, eIFs 1, 2, and 5, or small ribosomal proteins, copurified with the eIF3b immune complexes with efficiencies too low to make any reliable conclusions (data not shown).

Taken together, these results clearly demonstrate that stable expression of both eIF3c and eIF3a subunits is required for the overall integrity of the entire eIF3 holocomplex. They further imply that our following biochemical and phenotypic analysis of the eIF3c^KD^ cells must be regarded as the effect of the significant loss of the entire module ii accompanied by separation of modules i and iii from each other. Similarly, analysis of the eIF3a^KD^ cells must be viewed as the effect of the latter plus disintegration of module i and downregulation of module iii subunits.

### Downregulation of eIF3c and eIF3a but not eIF3j diminishes initiation rates and ceases cell growth.

**FIG 2:**
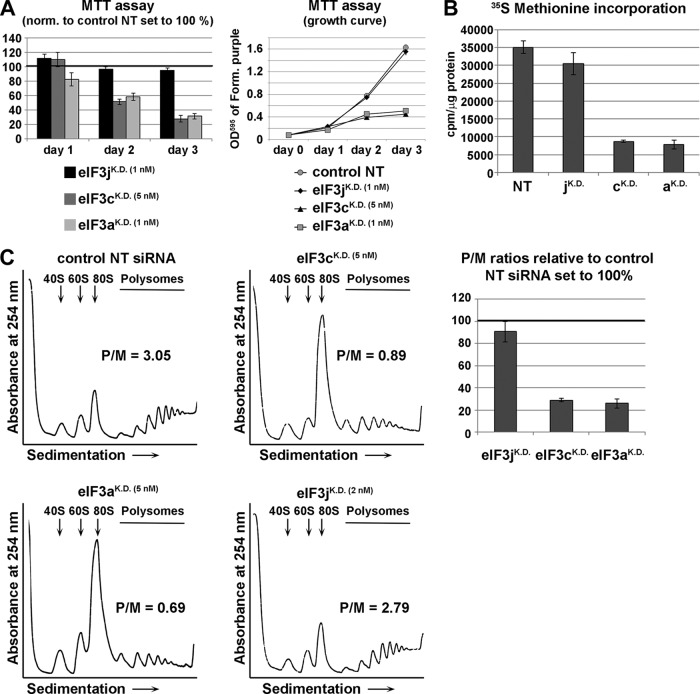
Downregulation of eIF3c and eIF3a but not eIF3j diminishes initiation rates and ceases cell growth. (A) The effect of knockdowns of eIF3j, eIF3c, and eIF3a using siRNAs at indicated concentrations on cell proliferation was assessed by the MTT assay 24, 48, or 72 h posttransfection in HeLa cells. Figures represent the results from three independent experiments ± SD. (B and C) The effect of knockdowns of eIF3j, eIF3c, and eIF3a using siRNAs at indicated concentrations on translation rates was assessed by the [^35^S]methionine incorporation (B) and polysome profile (C) analyses 72 h posttransfection. Positions of 40S, 60S, and 80S species are indicated by arrows. Graphs represent the results from three independent experiments ± SD. P/M, polysome-to-monosome ratio.

Visual inspection revealed that the morphology and viability of HeLa and HEK293 cells treated with eIF3j siRNA seemed unchanged compared to those of cells treated with control NT siRNA. However, the eIF3c^KD^ and eIF3a^KD^ HeLa cells became slimmer and with more pronounced spindle shape, and HEK293 cells showed a lower number and smaller size of starlike extensions barely stretching out of the cell body (data not shown). Importantly, we observed only slightly increased amounts of floating dead cells in either of the latter knockdowns compared to that of the NT control. Moreover, FACS analysis of cells stained with annexin V and a vital dye, Hoechst 33258, showed that the majority of HeLa and HEK293 cells were still alive and had not entered apoptosis 3 days posttransfection, in contrast to cells treated with bortezomib, which was used as an inducer of apoptosis (see Fig. S4 and S5 in the supplemental material). To further test the fitness of the siRNA-treated cells, we performed an MTT assay that is based on the reduction of the yellow-color MTT to blue formazan, a reaction that can be carried out only by metabolically active cells. Whereas the metabolic activity of the cell population in eIF3j^KD^ or control NT siRNA-treated cells was the same even after 3 days posttransfection, it decreased to 25 to 30% in both eIF3a and -c knockdowns (Fig. 2A). The cell number of both populations also decreased (down to ∼45% [data not shown]), indicating that both eIF3c^KD^ and eIF3a^KD^ cells slowed their growth rate and decreased metabolic activity compared to those of control cells.

Next, we analyzed efficiency of translation by performing the [^35^S]methionine incorporation assay in HeLa cells (Fig. 2B) and polysome profile analysis in both HeLa and HEK293 cells (Fig. 2C; see also Fig. S6 in the supplemental material). Compared to the NT siRNA-treated cells, eIF3j^KD^ produced only a minor (∼10 to 15%) drop in the methionine assay and just a modest reduction in the polysome-to-monosome (P/M) ratio (the latter was observed only in HeLa cells, not in HEK cells), clearly indicating that ∼5-fold reduction in the eIF3j protein levels has only a subtle, if any, effect on protein synthesis and cell fitness. In contrast, both eIF3c^KD^ and eIF3a^KD^ in HeLa cells reduced [^35^S]methionine incorporation by ∼75% (Fig. 2B), and the P/M ratios dropped by ∼75% and ∼50% in HeLa (Fig. 2C) and HEK293 cells, respectively (see Fig. S6 in the supplemental material) due to a marked shift of 80S couples from polysomes to a single monosome peak, which is a hallmark of impaired translation initiation rates. These findings clearly demonstrate the essentiality of both eIF3a and eIF3c subunits for translation initiation.

### eIF3 module i and to a lesser extent also module iii are capable of binding to the 40S ribosome on their own *in vivo*.

**FIG 3:**
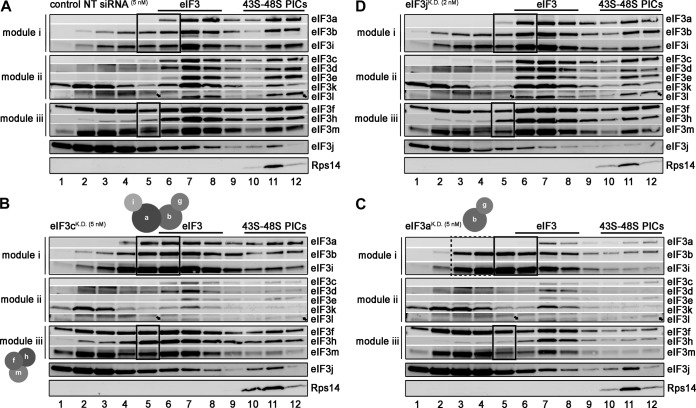
eIF3 module i is capable of binding to the 40S ribosome on its own. HeLa cells transfected with control NT siRNA (A) and siRNAs against eIF3c (B), eIF3a (C), and eIF3j (D) were treated with 0.2% formaldehyde (72 h posttransfection), and the WCEs were separated on 7 to 30% sucrose gradients. Collected fractions were subsequently subjected to Western blot analysis. These experiments were performed three times; for quantifications and SD, see Fig. S7 in the supplemental material. For description of boxes, see the text. Arrows indicate the eIF3l-specific band.

Next we took advantage of the fact that the eIF3c knockdown significantly reduces the amounts of module ii subunits resulting in separation of eIF3 into intact module i and most likely also module iii (recall that all of what is left of module ii most probably occurs in the 12-subunit eIF3 holocomplex [Fig. 1C]) and asked whether the separated modules i and iii are capable of 40S binding on their own. To do that, we treated the siRNA-transfected cells with 0.2% formaldehyde, ran the whole-cell extracts (WCEs) on 7 to 30% sucrose gradients, and collected 0.6-ml fractions from the top down to the section containing the 43S-48S PICs, similarly as was previously described for yeast cells (39). Thus, obtained fractions were subjected to Western blot analysis after reverse cross-linking. In control NT cells, a clear peak of the signal for all eIF3 subunits as well as for eIF3j can be observed in fractions 10 to 12 containing 40S subunits, which were visualized with an antibody raised against small ribosomal protein Rps14 (Fig. 3A). Note that 40S-free eIF3 sediments predominantly in fraction 7 (partly also in fractions 6 and 8), but 40S-free eIF3j runs mostly in the top 4 fractions.

In the eIF3c^KD^ cells, 40S binding of modules i and iii was reduced to ∼65% and ∼35%, respectively, compared to the control NT cells, whereas the 40S-associated amounts of module ii dropped down to ∼15%; note that the majority of what was left of module ii sedimented in fraction 7, where 40S-free eIF3 runs (Fig. 3B; see also Fig. S7A in the supplemental material). Binding of eIF3j was not affected. Considering that three eIF3 subspecies exist in eIF3c^KD^ cells as proposed above, these results suggest that (i) a major proportion of module ii indeed occurs associated with eIF3, a minor proportion of which is bound to 40S subunits (∼15% binding thus corresponds to binding of the intact eIF3 holocomplex), (ii) module i retains relatively high 40S binding affinity on its own (subtracting the amount corresponding to the intact eIF3 holocomplex bound to 40S ribosomes [15%] from the total 65% of the 40S-associated module i suggests that the solitary module i retains ∼50% of the 40S binding affinity of the intact eIF3 [40S-free module i accumulates in “boxed” fractions 5 and 6]), and (iii) module iii can also interact with 40S ribosomes separately from the rest of eIF3 although with markedly weaker affinity than module i (from the overall 35% of module iii occurring in 40S-containing fractions, approximately 20% [after subtracting ∼15% binding corresponding to the intact eIF3 holocomplex] seems to correspond to solitary module iii [40S-free module iii accumulates predominantly in the “boxed” fraction 5]). In fact, increased accumulation of all module iii subunits in fraction 5 provides the aforementioned indication that they all stay bound together in the eIF3c^KD^ cells. (Indeed, no conclusion can be drawn about the 40S binding of module ii from these experiments.)

The eIF3a^KD^—resulting in further disintegration of module i and downregulation of module iii subunits (Fig. 1C)—then markedly reduced 40S binding of all module i subunits along with subunits of the other two modules, as expected (Fig. 3C; see also Fig. S7B in the supplemental material). They were all down to ∼10 to 15% with the exception of eIF3b (down to ∼25%), which was previously shown to interact with the eIF3j-bound 40S subunits on its own (40). Since the distribution of module ii in the gradients was practically unchanged, we conclude that all we see in the 40S fractions corresponds to the intact 12-subunit eIF3 (with the exception of eIF3b). Note that compared to eIF3c^KD^, individual subunits of module i accumulate further up in “dash-boxed” fractions 3 and 4, clearly showing that module i no longer holds together and thus loses its 40S binding affinity. Also notice that whereas the “40S-free eIF3” fractions 7 and 8 contain relatively unchanged amounts of module iii subunits, the “boxed” fraction 5, containing “free module iii” in the eIF3c^KD^, is dramatically depleted of all three proteins in the eIF3a^KD^ cells, suggesting that the majority of module iii, which assembles from the reduced pool of its subunits in the eIF3a^KD^ cells, is immediately used to build the eIF3 holocomplex. And finally note that virtually complete disassembly of eIF3 had no effect on 40S association of eIF3j. This suggests that eIF3j is functionally and perhaps also structurally independent of the eIF3 holocomplex in living cells.

As mentioned above, earlier mostly *in vitro* studies suggested that eIF3j critically promotes eIF3 association with the 40S ribosome (28–30). We show here, however, that knocking down eIF3j in living cells has no effect on cell proliferation, and the initiation rates are impacted only very marginally (Fig. 2). It was therefore critical to examine the effect of the eIF3j^KD^ cells on 40S binding by eIF3 *in vivo*. As shown in Fig. 3D and in Fig. S7C in the supplemental material, we observed a small reduction (by ∼20%) in 40S association of a, d, e, and m subunits of eIF3 but not of the others, despite the fact that eIF3j amounts associated with 40S subunits were reduced by ∼80% in the eIF3j^KD^ cells. However, since our co-IP experiments showed that eIF3j^KD^ has no effect on the eIF3 integrity (Fig. 1C), we think that the observed effects are of a marginal significance, within an experimental error. With that said, we conclude that if eIF3j promotes eIF3 binding to 40S subunits *in vivo*, it would be to a degree undetectable by this technique.

### eIF3 module i significantly contributes to the eIF3 role in mRNA recruitment to 43S PICs *in vivo*.

**FIG 4:**
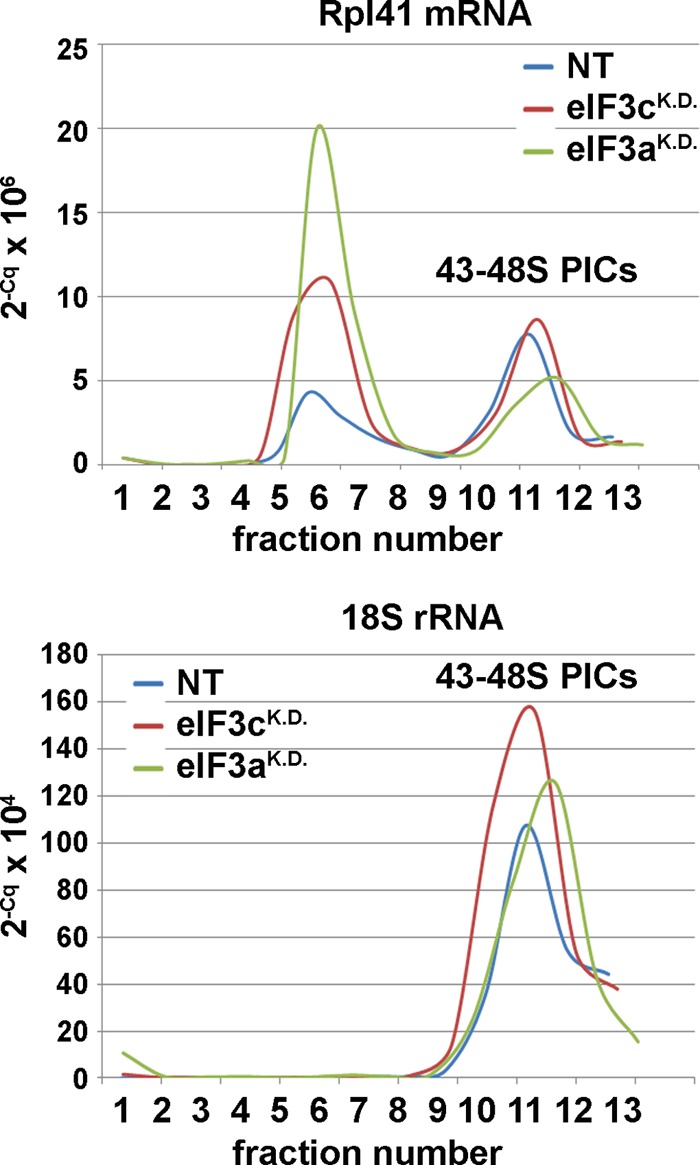
eIF3 module i significantly contributes to the eIF3 role in mRNA recruitment to 43S PICs *in vivo*. HeLa cells transfected with control NT siRNA and siRNAs against eIF3c and eIF3a were harvested without formaldehyde treatment 72 h posttransfection, and the WCEs were separated on 7 to 30% sucrose gradients. Total RNA was isolated from all collected fractions, and amounts of *RPL41* mRNA associated with native 48S PICs (visualized by 18S rRNA) were measured using real-time PCR. The amounts of mRNA were calculated as 2^−Cq^ × 10^6^ for *RPL41* mRNA and 2^−Cq^ × 10^4^ for 18S rRNA, where “2” represents the PCR efficiency and Cq the quantitation cycle.

With the eIF3c^KD^ predominantly reducing the amounts of eIF3 modules ii and iii on the 40S ribosome, and with the eIF3a^KD^ virtually eliminating 40S binding of eIF3, we asked whether or not mRNA recruitment to 43S PICs in these cells is also affected. Therefore, we measured the amounts of *RPL41* mRNA associated with native 48S PICs in WCEs of siRNA-transfected and control cells that were resolved on sucrose gradients as described above. We used *RPL41* mRNA since it is a well-established model mRNA in yeast systems (21). It is rather short and thus minimizes the “contamination” effects of large mRNAs assembled into mRNPs that often run in heavy sucrose fractions like ribosomal species and could lead to serious misinterpretations of the mRNA recruitment data. Using real-time PCR to monitor the presence of *RPL41* mRNA and 18S rRNA in 13 collected fractions as described before (23), we found that the ratio (%) of free to 40S-bound *RPL41* mRNA was about 40:60 in control NT cells (Fig. 4). The eIF3c^KD^ reverted this ratio to 60:40, indicating an underutilized pool of free mRNPs, and eIF3a^KD^ then further reduced (by ∼40% relative to the eIF3c^KD^) the 40S-bound amounts of *RPL41* mRNA, increasing the mRNA free/bound ratio to 75:25 (this experiment was repeated three times). These results suggest that module i is to a certain degree able to promote mRNA binding to 40S ribosomes on its own; however, to achieve optimal mRNA recruitment rates, it most likely requires additional support from module ii.

### Functional analysis of eIF3c and eIF3j knockdowns.

**FIG 5:**
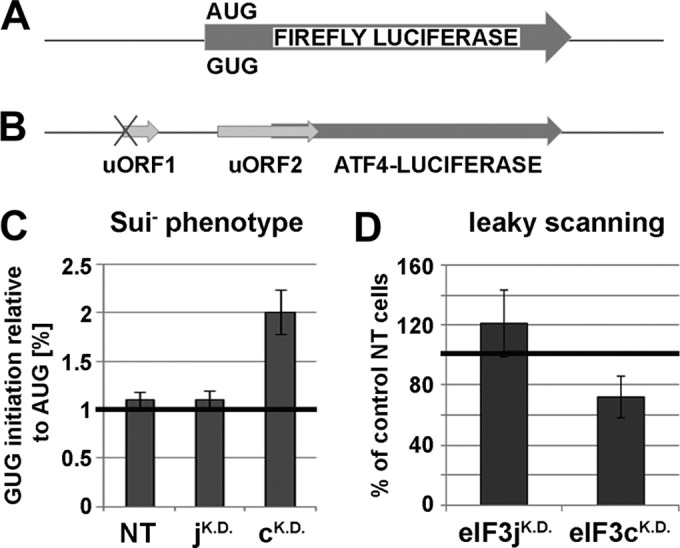
The eIF3c^KD^ relaxes the stringency of the AUG start codon selection *in vivo*. (A) Schematic of the firefly luciferase reporter with alternative start sites AUG or GUG. (B) Schematic of the ATF4-firefly luciferase reporter showing positions of its two short uORFs, the first of which is mutated at the AUG start site and the other naturally extends into the coding region. (C) Quantification of the Sui^−^ phenotype. HeLa cells were transfected with the indicated siRNAs, and 48 h later, the second round of transfection with individual reporters (AUG or GUG) together with a plasmid expressing the Renilla luciferase (used for normalization) was carried out. Luciferase activities were measured in WCEs 24 h after the second transfection. The mean percentages ± SD of the GUG initiation rates relative to those of AUG were calculated from three experiments. (D) Quantification of the leaky scanning phenotype. Same as described for panel C, except that the ATF4 reporter was transfected during the second round of transfection. The mean percentages ± SD of the increased or decreased skipping of the AUG start site of uORF2 in cells with knocked-down expression relative to control NT cells were calculated from three experiments.

Specific mutations in budding yeast orthologs of eIF3c and eIF3j, NIP1 and HCR1, produce phenotypes implicating these two subunits in regulation of stringent selection of the AUG start codon *in vivo* (32, 41). They either allow selection of a near-cognate codon to AUG (such as UUG or AUU) as the start codon (this is called a Sui^−^ phenotype and indicates a relaxed stringency of the start codon selection) or have a tendency to skip initiating AUG and continue scanning (this is called leaky scanning and indicates an inability to recognize AUG as the start codon [increased stringency]). Therefore, it was tempting to test the effects of knocking down the expression of eIF3c and eIF3j on accuracy of AUG recognition. To do that, we employed two luciferase reporters: one contained GUG in place of the luciferase AUG start codon (Fig. 5A), and the other contained short upstream ORF (uORF2) extending into the coding sequence of the ATF4 gene fused with luciferase (34) (Fig. 5B; only those 40S ribosomes that will skip the AUG of uORF2 will translate the luciferase gene). Relaxed stringency of the start codon selection should increase the luciferase activity with the GUG construct and reduce natural leaky scanning on the uORF2 construct, whereas increased stringency should increase leaky scanning and perhaps also reduce the activity of the GUG construct. As shown in Fig. 5C and D, whereas eIF3j^KD^ did not reveal any significant defects with either of the reporters, eIF3c^KD^ exhibited increased activity with the GUG construct (Sui^−^ phenotype) (Fig. 5C) and modest reduction in leaky scanning with the uORF2 construct (Fig. 5D). Since the mRNA levels of all reporters were unchanged in cells with knocked-down expression and control cells (data not shown), we conclude that eIF3c^KD^ has a detectable impact on the accuracy of AUG recognition.

## DISCUSSION

**FIG 6:**
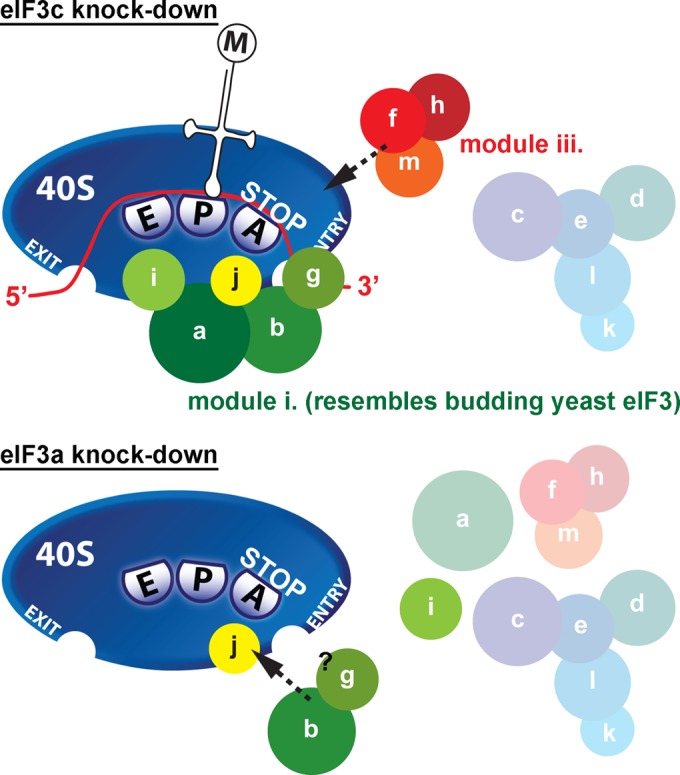
Schematics illustrating the effects of eIF3c and eIF3a knockdowns on integrity of the human eIF3 holocomplex and its ability to stimulate formation of the 48S PICs. The separate, intact eIF3 module i, resembling the budding yeast 5-subunit eIF3, is to a certain degree capable of stimulating formation of the 48S PICs. The 40S binding ability of the separate eIF3 module iii is limited. eIF3j associates with 40S ribosomes independently of the eIF3 holocomplex. The eIF3b subunit, perhaps still in complex with eIF3g, shows a weak 40S binding affinity on its own. For further details, see the text.

In this first (to our knowledge) complex *in vivo* analysis of human eIF3, we knocked down expression of its eIF3c and eIF3a subunits, which are both part of the octamer core of eIF3, and of eIF3j in two human cell lines and systematically analyzed their phenotypic and biochemical effects, following the fate of all 13 eIF3 subunits by several newly established techniques, which previously proved to be extremely useful in characterizing the translation initiation process in S. cerevisiae. We show that downregulation of eIF3j has no effect on cell proliferation of HeLa and HEK293 cells, as it only modestly reduces translation initiation rates without an apparent impairment of the 43S PIC assembly, in contrast to earlier suggestions. The deleterious eIF3c knockdown severely reduced protein but not mRNA levels of other module ii subunits (d, e, k, and l) and disintegrated the octamer core of eIF3, producing two separate modules, i and iii, apparently without interfering with their self-integrity (summarized in Fig. 6), which provides critical *in vivo* evidence for the bridging role of eIF3c proposed in earlier studies. The separate module i, composed of the “yeast core” a, b, g, and i subunits, preserved relatively high 40S binding affinity and an ability to promote mRNA recruitment to 40S subunits *in vivo* but proved to be defective in the proper AUG start codon selection. The eIF3a knockdown showed an effect identical to that of the eIF3c knockdown, and on top of that it disintegrated module i, severely impaired mRNA recruitment, and dramatically reduced protein levels of all but b, g, and i subunits (Fig. 6). Even though both HeLa and HEK293 cells are derived from completely different cellular precursors and thus might hold unique features due to their transformed nature, the fact that all tested knockdowns produced similar results in both cell lines suggests that the reported observations might be generalizable for at least human if not mammalian eIF3.

With respect to dramatically reduced protein levels in eIF3a and eIF3c knockdowns, Zeng et al. recently showed that eIF3m knockdown also specifically reduced expression of the remaining module iii subunits (f and h) and also that of eIF3c (other module ii subunits were not tested) but none of the module i subunits (38). These data, combined with ours and an earlier work by Zhang et al. (42), suggest mutual multiple-level regulation of gene expression of all eIF3 subunits that is critical for eIF3 integrity. The impact of the eIF3a knockdown on the abundance of most of the other eIF3 subunits observed here seems to be, to our knowledge, by far the broadest, suggesting that eIF3a could act as one of the main regulators in this process. This may in turn make this subunit a potent target in prospective anticancer therapies. A similar promise has been recently made also for the eIF3c subunit, since the genetic manipulations with its expression in five different cancer cell lines led to cell death (43).

Despite many attempts of several groups, the overall structure of eIF3 remains somewhat obscure (7, 14, 15, 44). It seems evident that the eIF3 core is formed by the PCI/MPN octamer to which other subunits bind (14–16). Our *in vivo* data strongly suggest, however, that within this octamer, the originally proposed modularity of eIF3 (10) can still be recognized. In WCEs of eIF3c knockdown cells, the anti-eIF3b antibodies pulled down all module i subunits, including eIF3a, one of the key members of the octamer, and module iii also seemed to be preserved intact. The eIF3a knockdown, which in addition disintegrated module i, produced merely the eIF3b-g dimer, indicating that the trimeric complex between the b, i, and g subunits is, in the absence of eIF3a, either not stable or does not form at all. The latter option gains significant support in a recent work by Dong et al. (24), reporting a detailed protein-protein interaction map for all module i subunits (a, b, i, and g), which suggested that binding of eIF3i to eIF3b is bridged by eIF3a and thus human eIF3i does not directly associate with the eIF3b-g dimer. On the other hand, this and our findings seem at odds with the most recent model of human eIF3 (14), the fact that formation of a stable trimer was observed *in vitro* (15, 28), and with the well-established protein linkage map of yeast eIF3 (18). To reconcile these somewhat contradicting observations, it could be proposed that eIF3i and -g subunits do interact with each other like in the budding yeast; however, this interaction is weaker than individual binding of eIF3i to eIF3a and eIF3g to eIF3b subunits, so only the b-g dimer sustained the stringency of our *in vitro* manipulations in eIF3a^KD^. Certainly, further work is needed to resolve these inconsistencies.

In any case, the model presented in Fig. 1A represents our attempt to integrate the data reported here and elsewhere into the likely spatial arrangement of all eIF3 subunits and their associated proteins. We propose that both eIF3a and eIF3c subunits are together critical for formation of the eIF3 octameric core, like in Neurospora crassa (45). In addition, eIF3c serves as a nucleation center for all module ii subunits (octameric e, k, and l, and nonoctameric eIF3d) and as a main attachment site for the all-octameric module iii. eIF3a then ensures attachment of eIF3i and the eIF3b-g dimer, with which it forms a stable module i.

Module i can eventually exist freely in the cytoplasm, if the bridging role of eIF3c, originally proposed by Zhou et al. (10), is compromised. An arrow between the i and g subunits in Fig. 1A indicates a probable contact between these two proteins as discussed above. A firm attachment of the entire module i to the octamer is highly likely further supported by the mutual contact between eIF3b and eIF3c subunits, as observed by Yahalom et al. (46). Existence of a stable module i is in good agreement with a pioneering attempt to reconstitute the eIF3 complex lacking eIF3c *in vitro*, which resulted in purification of the a, b, g, and i complex with reduced or completely eliminated amounts of other eIF3 subunits (12).

The arrangement of module ii within the octamer, as proposed in our model, is in accord with two recent findings by Imataka's et al. They revealed that eIF3 lacking the eIF3l subunit can be reconstituted, and together with eIF3l it lacks only the k subunit, whereas reconstituted eIF3 lacking either the k or d subunits is otherwise intact (12, 47). As for module iii, all module iii subunits were shown to form a stable heterotrimer *in vitro* (10) and very recently also *in vivo* (38), and the eIF3h subunit was shown to interact with eIF3c (10, 44). Indeed, we cannot rule out that one or more module iii subunits also contact the other major scaffolding subunit, eIF3a, as recently proposed (14). It could in fact explain why eIF3a and not eIF3c knockdown reduced expression levels of all module iii subunits (Fig. 1B). However, our coimmunoprecipitation data carried out without (Fig. 1C) or with (data not shown) cross-linking by HCHO as well as our sucrose gradient analysis (Fig. 3) strongly suggest that if these contacts really exist, they are most probably not strong enough to keep modules i and iii together in the absence of eIF3c.

Our observations that eIF3j^KD^ in both HeLa and HEK293 cells produced only a minor drop in the methionine incorporation assay and a modest reduction in the polysome-to-monosome (P/M) ratios and had no significant effect on the integrity of eIF3 as well as of the 43S PICs are not fully consistent with earlier studies, proposing a critical role for eIF3j in anchoring eIF3 to the 40S ribosome with a potential to also significantly influence mRNA recruitment (28–30). Hence, either only a minor proportion of the eIF3j protein from its overall cellular pool is sufficient to carry out these critical initiation functions of eIF3j or its importance deduced mostly from *in vitro* experiments was somewhat overestimated. In support of the latter, Sun et al. recently reported that the addition of eIF3j to either recombinant or native 12-subunit eIF3 did not increase its affinity for 40S subunits in native gels (7). Also, deletion of its budding yeast ortholog *hcr1* produces “only” a slow-growth phenotype (31, 32) that is, in addition, caused by its defect in termination and stop codon readthrough rather than in initiation (3). Hence, it seems highly likely that also the role of human eIF3j in translation is not critical but only stimulatory. Furthermore, given the fact that eIF3j is often missing from the purified 12-subunit complex (12, 14, 16, 30, 48, 49) and even a complete disassembly of eIF3 in eIF3a^KD^ had no effect on its association with 43S PICs (Fig. 3), it remains to be investigated whether eIF3j really is a bona fide eIF3 subunit or just an eIF3-associated factor.

In contrast to eIF3j^KD^, both eIF3c^KD^ and eIF3a^KD^ dramatically affected both translational rates as well as the cellular growth. It was expected in the case of eIF3c^KD^, because reconstituted eIF3 lacking eIF3c (and other subunits due to that; see above) was shown earlier to exhibit very little activity in a toe print assay monitoring the efficiency of formation of initiation complexes on the AUG start codon (12). In the case of eIF3a^KD^, the expectations were not so obvious, because Dong et al. showed earlier that downregulating the eIF3a level in HeLa cells by transient transfection of antisense eIF3a cDNA reduced the [^35^S]methionine incorporation only by 25% and levels of other eIF3 subunits remained the same (50). Despite the fact that they achieved a relatively similar degree of eIF3a downregulation as we did here, our eIF3a^KD^ data are in sharp contrast with Dong et al.'s findings. We propose that these obvious discrepancies could arise from either different experimental approaches chosen to knock down eIF3a or from a possibility that there is a relatively low threshold of eIF3a expression, below which the effects documented here can only be observed.

Finally, our observations suggest that module i promotes mRNA binding to 40S ribosomes on its own to a certain degree, but to achieve a maximal efficiency, it requires additional support from module ii (Fig. 4). These data are in a good agreement with several recent observations: (i) eIF3a (module i) and eIF3c (module ii) were shown to contain RNA binding domains capable of RNA binding *in vitro* (7); (ii) module ii subunits eIF3c, -d, and -e interact with eIF4G, which is known to promote mRNA loading onto the 40S ribosome (26) (Fig. 1A); (iii) yeast homologues of eIF3a and -c, TIF32 and NIP1, were also proposed to play a major role in mRNA binding to the 43S PICs *in vivo* (21–23), suggesting evolutionary conservation of the function(s) of these two eIF3 subunits. In addition to that, the eIF3c^KD^ negatively impacted accuracy of AUG recognition *in vivo* (Fig. 5), which is also consistent with the yeast data, where both NIP1 and TIF32 were implicated in regulating the stringency of start codon selection in close cooperation with their binding partners eIF1, eIF2, and eIF5 (21, 41, 51). Likewise, eIF1 together with eIF1A (both key regulators of AUG recognition) interact with eIF3c and eIF3a also in human (14) (Fig. 1A). Together, these findings clearly demonstrate that module i and eIF3c in principle represent the budding yeast eIF3 complex with its basic functions. It may be therefore suggested that a, b, c, g, and i subunits constitute some sort of a primordial eIF3 that gradually evolved by adding more subunits with extra functions to cope with increasing complexity of life, with the striking and rather mysterious exception of budding yeast S. cerevisiae.

## Supplementary Material

Supplemental material
