# Supplementary material for: Functional and Biochemical Characterization of Human Eukaryotic Translation Initiation Factor 3 in Living Cells
Source: Mol Cell Biol. 2014 Aug;34(16):3041–52. doi: 10.1128/MCB.00663-14 (PMC4135593; doi:10.1128/MCB.00663-14)
Supplement: Supplemental material [file supp_34_16_3041__index.html]

Supplemental material 

# Functional and Biochemical Characterization of Human Eukaryotic Translation Initiation Factor 3 in Living Cells

## Supplemental material

**Files in this Data Supplement:**

- Supplemental file 1 -

  Fig. S1 (eIF3c and eIF3a in expression of module ii and iii subunits of eIF3), S2 (Effect of knockdown of eIF3c and eIF3a), S3 (eIF3c linkage of eIF3 modules), S4 and S5 (Apoptosis after transfection with siRNA against eIF3j, eIF3c, or eIF3a), S6 (Effect of knockdown of eIF3j, eIF3c, and eIF3a), and S7 (Module i binding to the 40S ribosome) and Table S1 (Sources of antibodies used)

  PDF, 575K
