# Supplementary material for: Functional and Biochemical Characterization of Human Eukaryotic Translation Initiation Factor 3 in Living Cells
Source: Mol Cell Biol. 2014 Aug;34(16):3041–52. doi: 10.1128/MCB.00663-14 (PMC4135593; doi:10.1128/MCB.00663-14)
Supplement: Supplemental material [file MCB.00663-14_zmb999100548so1.pdf]

SUPPLEMENTARY FIGURES AND FIGURE LEGENDS

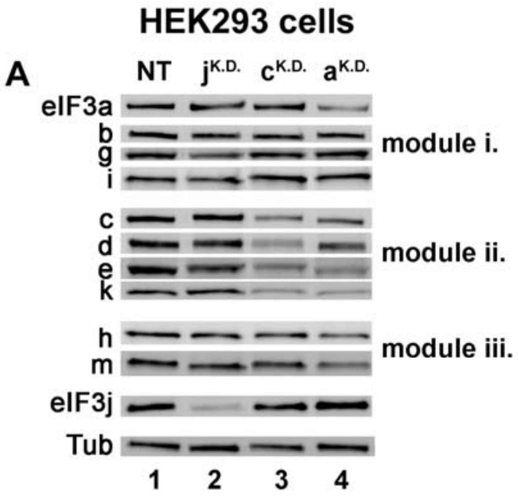

relative amounts normalized to Tub (NT set to 1)

	eIF3j$^{K.D.}$	eIF3c$^{K.D.}$	eIF3a$^{K.D.}$
a	**0.98** ± 0.06	**1.28** ± 0.15	**0.44** ± 0.13
b	**0.84** ± 0.07	**0.86** ± 0.14	**1.09** ± 0.20
g	**0.74** ± 0.19	**0.98** ± 0.19	**0.94** ± 0.12
i	**0.99** ± 0.12	**1.14** ± 0.23	**1.12** ± 0.10
c	**1.06** ± 0.12	**0.37** ± 0.04	**0.53** ± 0.14
d	**0.87** ± 0.05	**0.38** ± 0.04	**0.60** ± 0.05
e	**0.79** ± 0.05	**0.44** ± 0.15	**0.37** ± 0.12
k	**1.29** ± 0.28	**0.36** ± 0.09	**0.35** ± 0.14
h	**0.92** ± 0.16	**1.02** ± 0.20	**0.61** ± 0.16
m	**0.94** ± 0.06	**0.98** ± 0.01	**0.59** ± 0.14
j	**0.14** ± 0.03	**1.16** ± 0.18	**1.23** ± 0.27

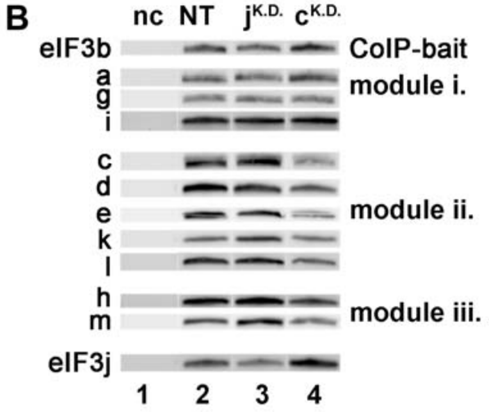

relative amounts normalized to eIF3b (NT set to 1)

	eIF3j$^{K.D.}$	eIF3c$^{K.D.}$
eIF3b	1	1
a	**1.21** ± 0.09	**1.07** ± 0.16
g	**0.96** ± 0.4	**1.23** ± 0.04
i	**0.93** ± 0.24	**1.19** ± 0.16
c	**0.83** ± 0.04	**0.47** ± 0.03
d	**1.00** ± 0.03	**0.63** ± 0.22
e	**1.05** ± 0.11	**0.65** ± 0.06
k	**0.99** ± 0.18	**0.77** ± 0.02
l	**1.09** ± 0.21	**0.62** ± 0.18
h	**1.16** ± 0.2	**0.70** ± 0.12
m	**1.10** ± 0.01	**0.61** ± 0.18
j	**0.50** ± 0.05	**1.08** ± 0.25

FIG S1. eIF3c and eIF3a control expression of module ii and iii subunits of eIF3 and link all three modules tightly together in HEK293 cells. (**A**) Effect of siRNA targeting eIF3j (2nM), eIF3c (5nM), and eIF3a (5nM) mRNAs on protein levels of eIF3 subunits and other eIFs determined by Western blotting 72 h post-transfection (this experiment was repeated numerous times with similar results). NT – control non-targeted cells; Tub – loading control, anti-TUBA4A from Sigma, cat. no. T6074. Quantifications were carried out essentially the same as in Figure 1B. (**B**) Effect of siRNA targeting eIF3j (2nM), eIF3c (5nM), and eIF3a (5nM) mRNAs on integrity of eIF3 determined by the co-immunoprecipitation assay using eIF3b as a Co-IP bait (anti-eIF3b antibody from Santa Cruz, cat. number sc-16377) followed by Western blotting 72 h post transfection (see also Fig. S2). nc - negative control (beads only – no antibody). Quantifications were carried out essentially the same as in Figure 1C.

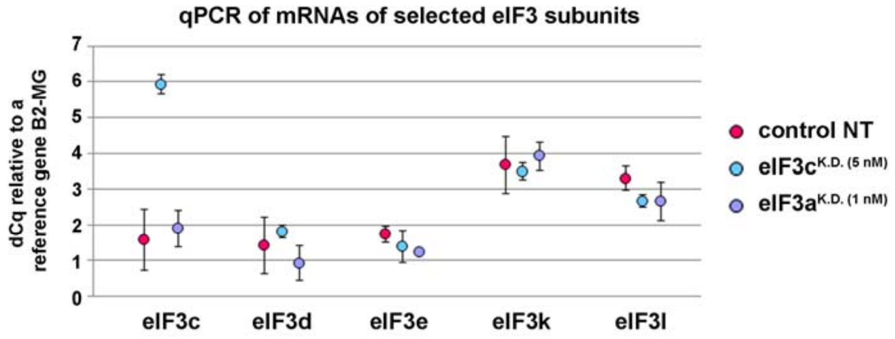

FIG S2. The effect of knock downs of eIF3c and eIF3a in HeLa cells using siRNAs (including control NT siRNA) at indicated concentrations on mRNA levels of selected eIF3 subunits was assessed by quantitative PCR 72 h post transfection. Figure represents the results of three independent experiments ± SD. The dC_q value displays the difference in the C_q value of the indicated eIF3 subunit (x-axis) with respect to the C_q value of the reference gene B2-MG: C_q(eIF3 subunit) – C_q(B2-MG). An increase in dC_q indicates a decrease in the mRNA level of a corresponding eIF3 subunit. Please note that only the targeted mRNA of eIF3c in the cells treated with siRNA against eIF3c is down-regulated. The mRNA levels of other eIF3 subunits are unchanged when compared to control NT cells.

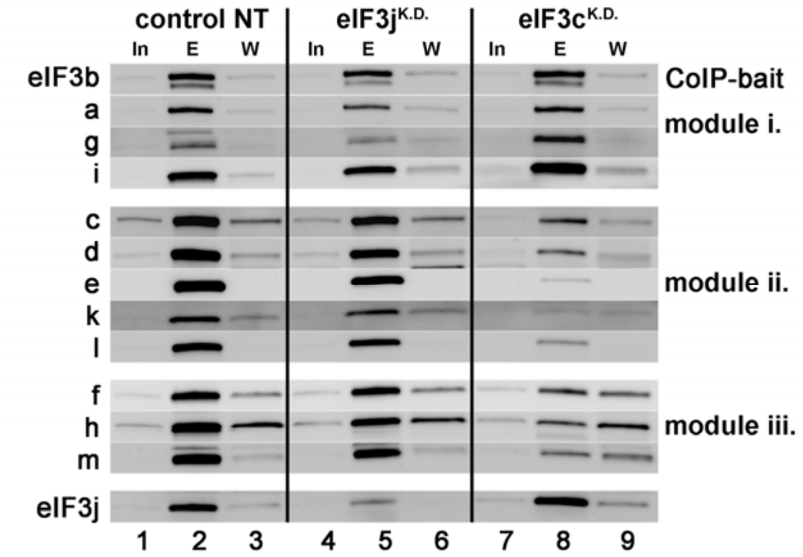

FIG S3. eIF3c links all three eIF3 modules together. Effect of siRNA targeting eIF3j (2nM) and eIF3c (5nM) on integrity of eIF3 determined by the co-immunoprecipitation assay using eIF3b as a CoIP bait (anti-eIF3b antibody from Santa Cruz, cat. number sc-16377) followed by Western blotting 72 h post transfection in HeLa cells. In – input (2.5%); E – elution (50%); W – wash (2.5%). Please note the enriched amounts of module iii subunits in the "Wash" fraction in the eIF3c$^{K.D.}$ (lane 9) strongly suggesting that majority of module iii no longer associates with module i.

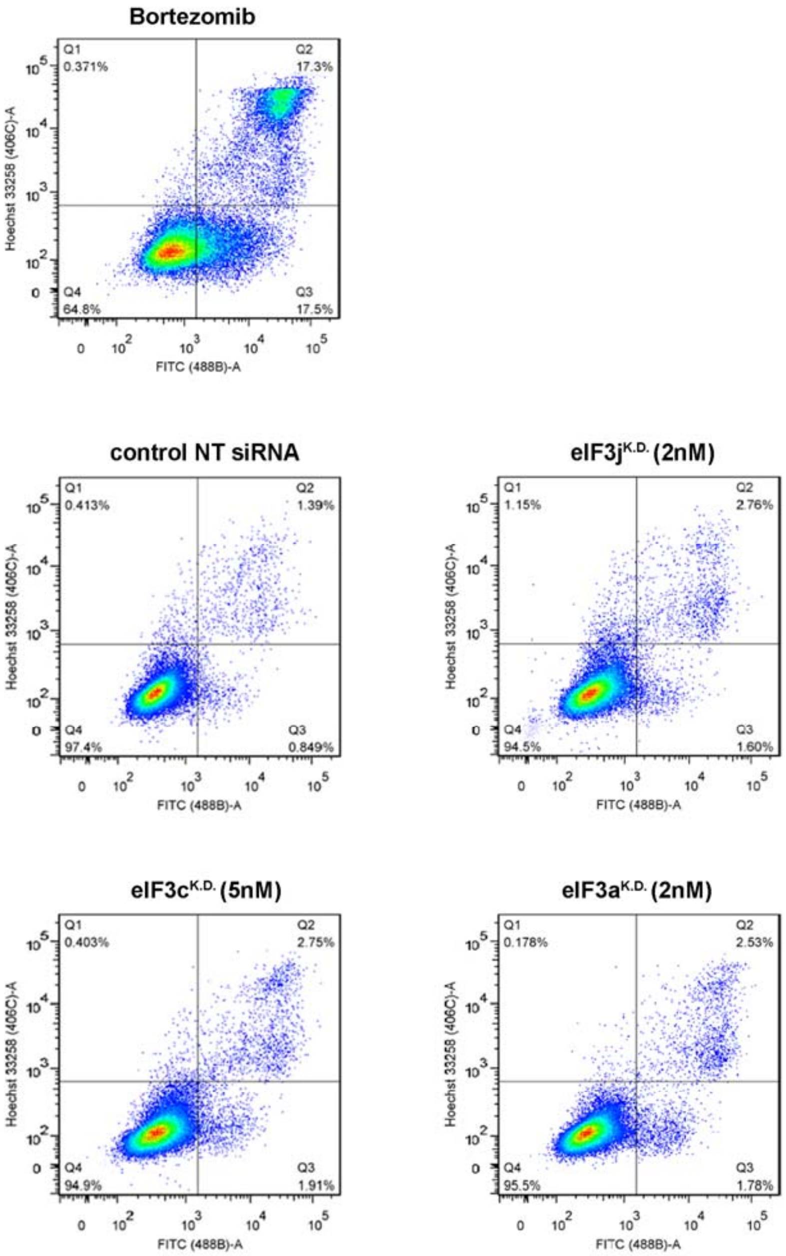

FIG S4. HeLa cells do not enter apoptosis after transfection with siRNA against eIF3j, eIF3c or eIF3a. FACS analysis of HeLa cells stained with Annexin V-FITC (X-axis) and Hoechst 33258 (Y-axis) to detect apoptosis was performed three days after siRNA transfection. Proteasome inhibitor Bortezomib (0.2 μM, 20 h-treatment) induced apoptosis in ~ 35% cells (17.3% early apoptotic cells (quadrant Q3) and 17.5% late apoptotic cells (quadrant Q2)). In contrast, 97% of control NT siRNA-treated cells (and similarly eIF3j, eIF3c or eIF3a siRNA-treated cells) were localized in quadrant Q4 and showed no signs of early or late apoptosis. Experiment was performed twice; data from a typical experiment are shown.

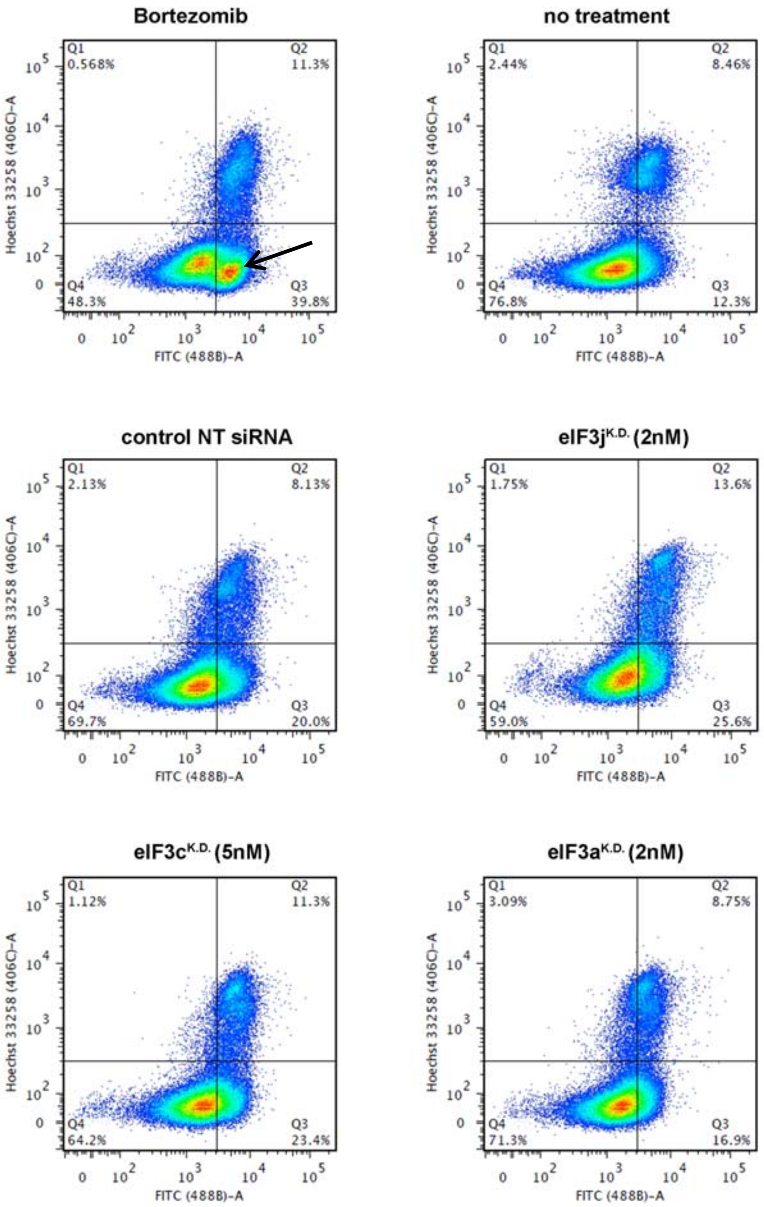

FIG S5. HEK293 cells transfected with siRNA against eIF3j, eIF3c or eIF3a do not show increased apoptosis. FACS analysis of HEK293 cells stained with Annexin V-FITC (X-axis) and Hoechst 33258 (Y-axis) to detect apoptosis was performed three days after siRNA transfection. Untreated cells served as a negative control (upper right panel); a population of cells stained with Hoechst 33258 and/or Annexin V-FITC present in quadrants Q2 and Q3 indicates cells damaged during the harvesting process that made possible the staining with Hoechst 33258 and Annexin V. Proteasome inhibitor Bortezomib (0.2 µM, 20 h-treatment; upper left panel) induced apoptosis in ~ 20% cells above control (sub-population in quadrant Q3, indicated by an arrow). None of the siRNA-treated cells exhibit this sub-population; indeed they are similar to the untreated and control NT siRNA-treated cells. At all, we conclude that the down-regulation of eIF3j, eIF3c or eIF3a do not induce apoptosis three days after transfection as there is no difference to control NT siRNA. Experiment was performed twice; data from a typical experiment are shown.

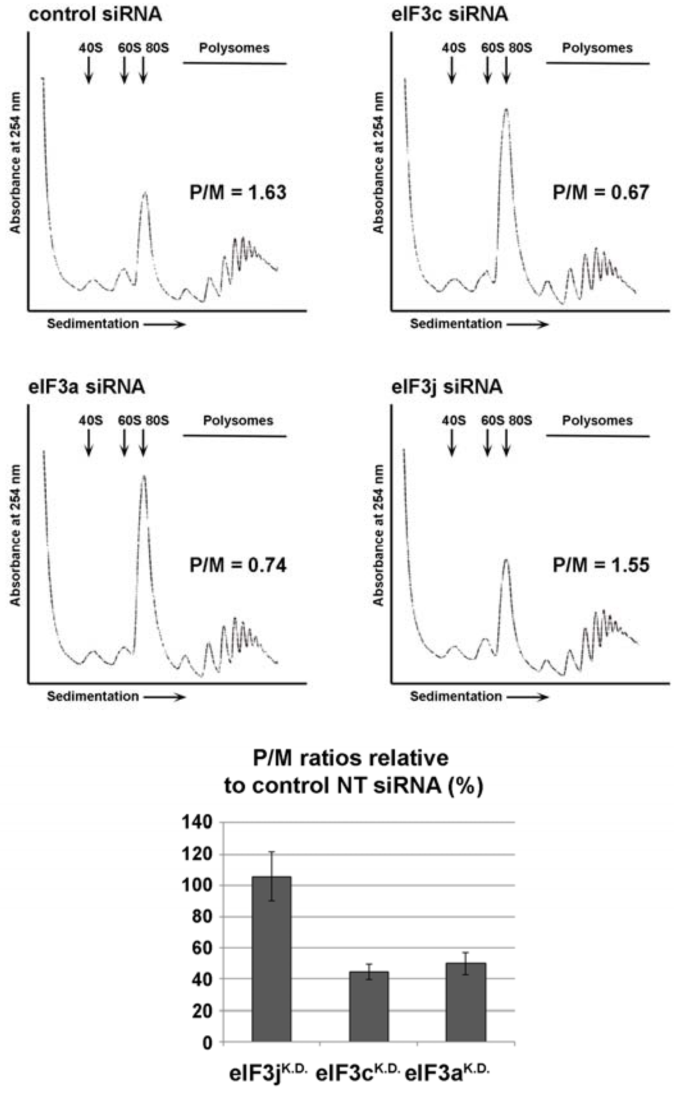

FIG S6. The effect of knock-downs of eIF3j, eIF3c and eIF3a using siRNAs at indicated concentrations on polysome profiles in HEK293 cells 72 h post transfection. Positions of 40S, 60S and 80S species are indicated by arrows. Graphs represent the results of three independent experiments ± SD. P/M – polysome to monosome ratio.

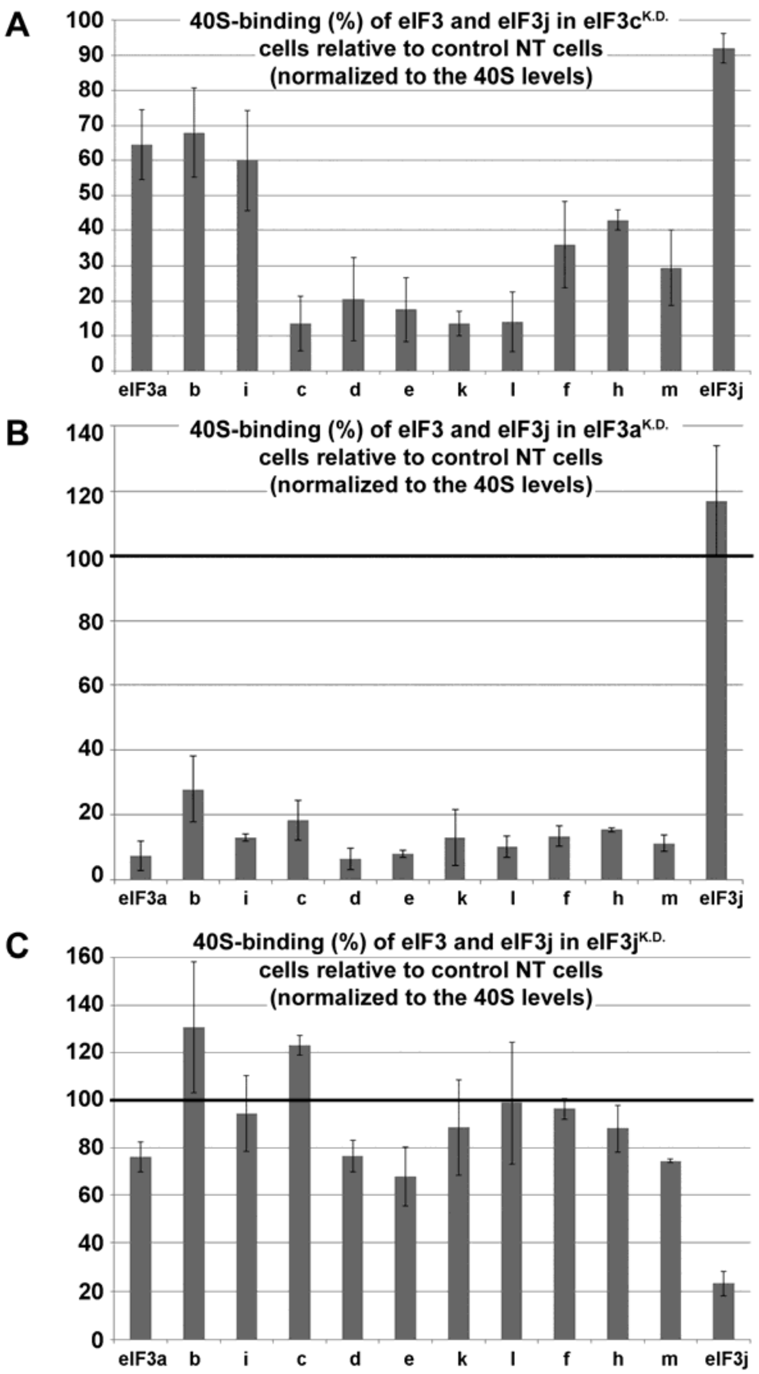

FIG S7. Module i is capable of binding to the 40S ribosome on its own – 40S-binding (%) of eIF3 subunits and eIF3j in eIF3c$^{K.D.}$ (*A*), eIF3a$^{K.D.}$ (*B*), and eIF3j$^{K.D.}$ (*C*) HeLa cells relative to control cells as shown in Figure 3. Proportions of the 40S-bound proteins relative to the amount of 40S subunits (RPS14 signal) were calculated using NIH ImageJ from three independent experiments. The resulting values obtained with the control NT cells were set to 100% and those obtained with eIF3c$^{K.D.}$ (*A*), eIF3a$^{K.D.}$ (*B*), and eIF3j$^{K.D.}$ (*C*) cells were expressed as percentages of control NT cells (SDs are given).

SUPPLEMENTARY TABLES

TABLE S1. The source of all antibodies used.

antibody	source
eIF3a	Cell Signaling # 2538
eIF3b (for Western blot)	Santa Cruz # sc-28857
eIF3b (for CoIP)	Santa Cruz # sc-16377
eIF3c	Santa Cruz # sc-28858
eIF3d	kind gift of Dr. Imataka
eIF3e	Abcam # ab36766
eIF3f	kind gift of Dr. Imataka
eIF3g	kind gift of Dr. Imataka
eIF3h	Cell Signaling # 3413
eIF3i	Sigma # HPA029939
eIF3j	Santa Cruz # sc-50356
eIF3k	Abcam # ab85968
eIF3l	kind gift of Dr. Imataka
eIF3m	Sigma # HPA031063
eIF2α	Santa Cruz # sc-11386
eIF5	Santa Cruz # sc-282
Rps14	Santa Cruz # sc-68873
α-Tubulin	Sigma # T6074
